# Schizophrenia versus Traumatic Brain Injury: A Diagnostic Challenge

**DOI:** 10.1192/j.eurpsy.2025.1847

**Published:** 2025-08-26

**Authors:** N. Monteiro, M. Abrantes, S. Morais

**Affiliations:** 1 Centro de Responsabilidade Integrada de Psiquiatria, Centro Hospitalar e Universitário de Coimbra (CHUC); 2 Faculdade de Medicina, Universidade de Coimbra, Coimbra, Portugal

## Abstract

**Introduction:**

Traumatic brain injury (TBI) is a rare, yet possible cause of psychosis (Fujii D *et al*. Psychiatr Clin North Am. 2014; 37(1):113-24), with one of the main challenges being distinguishing between Psychosis secondary to traumatic brain injury (PSTBI) and schizophrenia (SZ).

**Objectives:**

To discuss the diagnostic challenges in patients with psychosis and history of TBI.

**Methods:**

In addition to describing a case report of a male with psychotic symptoms presenting after a severe traumatic brain injury, research was undertaken in PubMed and other databases using the keywords “traumatic brain injury”, “psychosis” and “schizophrenia”.

**Results:**

Our patient is a 36 year-old male who suffered a severe TBI at age 22, with consequent frontal and temporal encephalomalacia. Initially he presented with persecutory delusions, delusional perceptions associated with colors, social isolation and decline in academic performance, which were attributed to Post-Concussion Syndrome. However, these symptoms would remain for years to come, leading to the new diagnosis of SZ, at age 25. This way, he started intramuscular antipsychotic medication, which reduced psychotic symptoms and improved his academic performance. This amelioration, at age 30, led to the belief in another diagnosis: Brief Psychotic Episode (after brain trauma). Consequently a reduction in antipsychotic dosage was tried but a resurgence of psychotic symptoms was observed at age 33, which led to the reintroduction of antipsychotic medication, and the reconsideration of the diagnosis of SZ. When we examined him, at age 36, he presented similar symptoms to those observed after the brain injury, intensified by years without antipsychotics. We also found that he had regular use of cannabinoids since age 16 and that his brother was diagnosed with Schizoaffective disorder.

**Image:**

**Figure:**
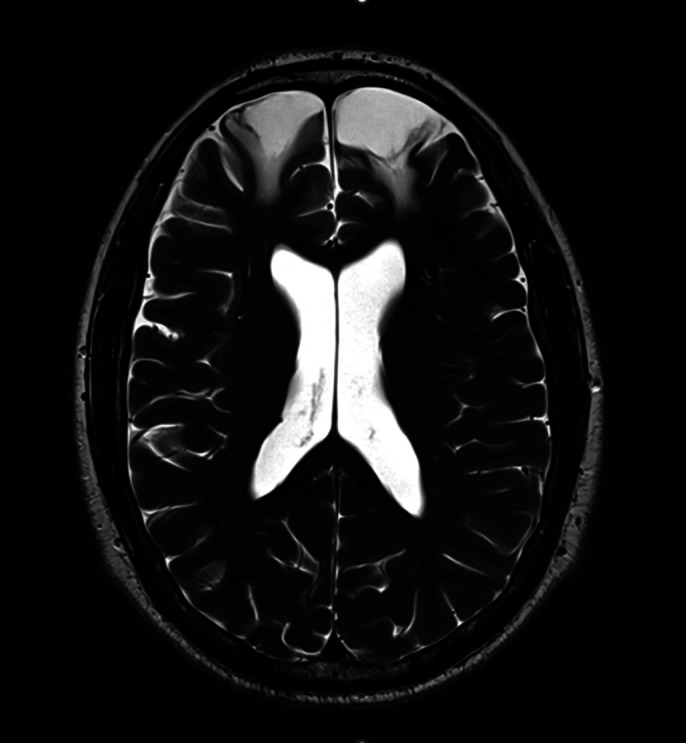

**Image 2:**

**Figure:**
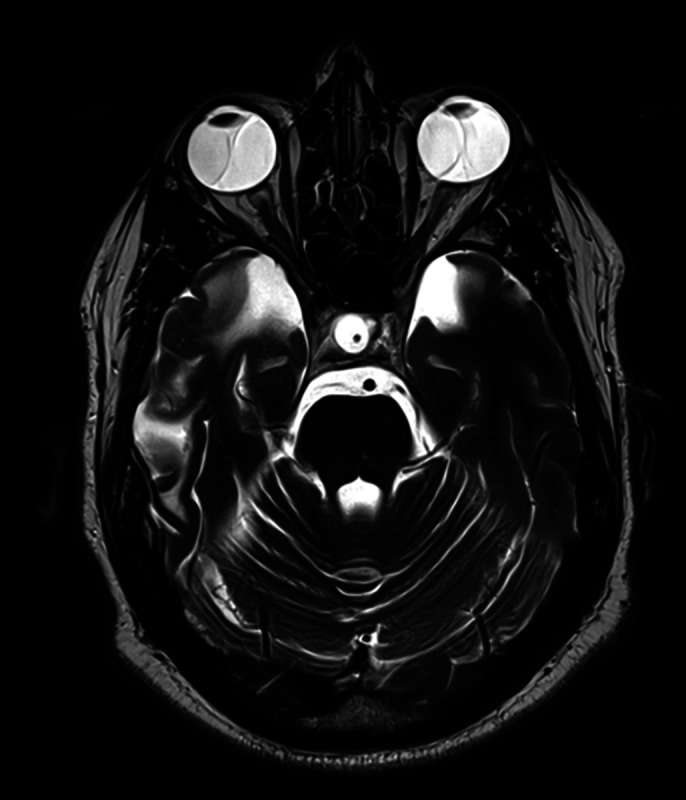

**Conclusions:**

PSTBI usually occurs after a TBI with frontal and temporal lesions, and psychotic symptoms like persecutory delusions, with the frontal lesions being a possible explanation for the decline in cognitive function by causing deficits in attention, executive functions and memory (Fujii D *et al*. Journal of Neuropsychiatry Clinics in Neuroscience 2002;14:130-140). SZ can similarly explain many of the findings presented, like psychotic symptoms, social isolation and decline in cognitive function due to negative symptoms, specially considering the use of cannabinoids and genetic vulnerability present in this patient and the fact that TBI is also a risk factor for the development of SZ. This case highlights the difficulty in the differential diagnosis between PSTBI and SZ, given that it presents aspects that can point in both directions.

**Disclosure of Interest:**

None Declared

